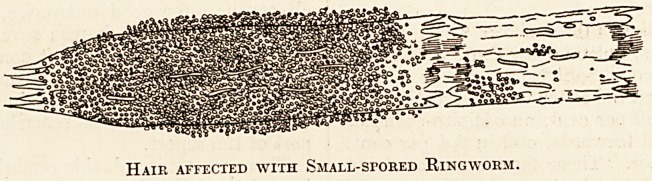# The Diagnosis of Ringworm of the Scalp

**Published:** 1908-05-09

**Authors:** 


### DERMATOLOGY.

### THE DIAGNOSIS OF RINGWORM OF THE SCALP.

Ringworm of the scalp is sometimes overlooked, and, on the other hand, patches of alopecia areata, or bald patches following a local impetiginous inflammation, are sometimes wrongly diagnosed as ringworm. Mistakes also arise as to when a case of ringworm is cured. The key to the diagnosis of ringworm of the scalp is the recognition of the diseased hair stump. Apart from this it is impossible to be sure whether a case is one of ringworm or not, and, moreover, it is impossible to know whether a case is cured or not.

Typical cases of ordinary small-spored ringworm are easily recognised by the presence of circumscribed rounded areas, in which the hairs are broken short like a field of stubble. The stumps look dull compared with the surrounding healthy hairs, and on passing the finger over the patch they feel velvety, and not bristly, as would hairs which had merely been cut short with scissors. It is found, too, that they have lost their elasticity, and that they remain sticking up in different directions after firmly stroking the patch with the finger or forceps, instead of flying back to their original position as normal hairs would do, or that they bend at right angles. On pinching up a bundle of these stumps between the finger and thumb they come away on the least traction, and without giving any pain to the patient. Most of the stumps in the bundle thus extracted have become broken towards the root end; a few come away with the soft bulb attached.

Hair affected with Small-spored Ringworm.

To confirm the diagnosis microscopically a drop of liquor potassæ is put on to the centre of a clean coverglass, and the latter is gently lowered on to a slide bearing a suspected hair. The specimen thus prepared is allowed to stand for five or ten minutes, and then examined under a $\frac{1}{4}$-inch or a $\frac{1}{6}$-inch objective. In the case of a small-spored ringworm the hair stump will be found to be jaggedly broken at each end; one end of the shaft will be comparatively free from fungus, but its surface will be denuded of its cuticle and eroded (a condition in itself practically diagnostic of small-spored ringworm). The other end of the shaft will be surrounded by a sheath made up of closely packed rounded spores of about a third or a quarter of the size of a red-blood corpuscle (see figure).

In an ordinary case of ringworm all this is straightforward and easy. But difficulty arises when, as is often the case, the ringworm patches are thickly covered with scales; or when the patches are not so sharply marked on account of the persistence of many long, healthy hairs on the affected areas; or in the case of a ringworm patch which has become partially bald as the result of active treatment, or of accidental inflammation of the patch, so that the question of diagnosis from alopecia areata arises; or when a case is apparently well, and one has to decide whether the child is quite free from infection or whether there are diseased stumps still present. The only way to be sure of these points is to become familiar with the broken ringworm stumps by examination with a lens. A watchmaker's lens is best, as it can be held in the eye, leaving free the hands. In every case of suspected ringworm of the scalp—and, indeed, in all cases of scalp affection in children—the lesions should be carefully examined under the lens, using a pair of forceps to turn aside the hairs, or to scrape up any scales in the search for such stumps as have been described above—*i.e.* stumps which are lustreless, non-elastic, and which come away on slight traction, breaking off in the follicle. It is not sufficient, however, to make a diagnosis of ringworm; if one patch is discovered the whole head should be carefully examined in order to determine the extent of the disease.

In regard to the diagnosis of ringworm from alopecia areata the following points must be noted: The smooth, shiny, perfectly bald circumscribed patch of alopecia areata contrasts strongly with the ringworm patch, which is not bald, but covered with broken stumps, and often with scales. The short hair stumps which may be present at the margin of an alopecia patch are quite characteristic and distinct from those of ringworm. They are thick and pigmented at the free end, and fine and pale at the attached end. They are translucent and elastic, and when pulled at they come away with a sharp click, their atrophied root being firmly but quite superficially attached. Even in the case of a ringworm patch which has become partially bald as the result of treatment, any ringworm stumps present can be easily distinguished from alopecia stumps if one is familiar with their characters as detailed above.

## Figures and Tables

**Figure f1:**